# Genetic Diversity and Biological Characteristics of a Novel Feline Calicivirus Strain Isolated From Zoo‐Captive South China Tiger (*Panthera tigris amoyensis*)

**DOI:** 10.1155/tbed/4900253

**Published:** 2026-10-09

**Authors:** YuShan Ding, Niu Zhou, Hai Wang, Zhaoyang Liu, Fen Shan, Minting Liu, Yajiang Wu, Chen Wang, Mengna Lv, Wanqing Li, Zujin Chen, Heng Liu, Tianping Li, Shiming Peng, Xuanjiao Chen, Weijun Xie, Xinyi Cao, Wu Chen, Yongyi Shen

**Affiliations:** ^1^ College of Veterinary Medicine South China Agricultural University Guangzhou China scau.edu.cn; ^2^ Guangzhou Zoo Guangzhou China; ^3^ Guangzhou Wildlife Research Center Guangzhou China; ^4^ Luoyang City Landscape Gardening Center LuoYang China; ^5^ Guangzhou University Guangzhou China gzhu.edu.cn; ^6^ School of Animal Sciences and Veterinary Medicine Hainan University Sanya Hainan China hainu.edu.cn; ^7^ School of Life and Health Sciences Hainan Province Key Laboratory of One Health Hainan University Hai kou Hainan China hainu.edu.cn

**Keywords:** genetic diversity, novel feline calicivirus, *Panthera tigris amoyensis*, phylogenetic analysis, VP1 protein

## Abstract

Feline calicivirus (FCV) is a common pathogen causing upper respiratory tract disease in domestic cats. Nonetheless, its threat and genetic features in endangered wild felids remain poorly understood. In this study, a novel FCV strain from a captive South China tiger (*Panthera tigris amoyensis*) was isolated, designated as FCV LYH1224. Viral RNA was detected in multiple tissues of the deceased tiger, including the heart, liver, spleen, lungs, and kidneys, suggesting broad tissue tropism and FCV contributed substantially to the fatal outcome. In vitro growth kinetics showed that the virus replicates efficiently in Crandell‐Rees feline kidney (CRFK) cells. Whole‐genome sequencing and phylogenetic analysis indicated that it was classified as genotype II (GII) and exhibited unique evolutionary lineages. Within GII, it clusters closely with the previously reported tiger‐derived strain FCV‐YH/16 but exhibits an independent branch in the VP1 gene tree, suggesting complex genetic variation among FCV strains from diverse wildlife origins. Sequence identity analysis indicated that FCV LYH1224 was genetically distant from vaccine strains F9 and 2280, with whole‐genome nucleotide identity of 76.6% and 77.0%, respectively. Notably, the VP1‐E region displayed the lowest identity, with nucleotide identity of 68.1% and 66.7%, respectively. Analysis of key amino acid residues revealed distinct substitutions within the neutralizing epitope (aa 445–451) and common mutations at virulence‐related positions 452A and 492I, which were absent in reference strains, suggesting potential antigenic diversity and host‐specific adaptations. This study provides a comprehensive genomic characterization of FCV strain in South China tigers, highlighting the genetic diversity and distinctive antigenic properties observed in this isolate compared with existing strains. The considerable genetic divergence from vaccine strains emphasizes the importance of ongoing surveillance and the inclusion of wildlife‐derived strains in vaccine development strategies.

## 1. Introduction

Feline calicivirus (FCV) is a highly contagious pathogen belonging to the genus *Vesivirus* of the Caliciviridae family, which poses a significant threat to feline health worldwide [1, 2]. FCV infection presents with various clinical manifestations, with the most common signs being acute upper respiratory tract disease and oral ulceration [3]. In some cases, particularly in young kittens, pneumonia may develop, resulting in dyspnea and systemic illness [4]. Recently, the global emergence of virulent systemic FCV (VS‐FCV) strains has been reported; these cause severe, often fatal multisystemic disease characterized by high fever, subcutaneous edema, cutaneous ulceration, alopecia, and necrosis involving multiple organs, including the liver, pancreas, and spleen [5–8]. FCV strains are classified into genotype I (GI) and genotype II (GII). GI strains are prevalent worldwide, whereas GII strains are predominantly observed in Asia [9, 10]. Recent surveillance in China has revealed significant genetic diversity, frequent recombination events within and between genotypes, and genetic differentiation between current field strains and established vaccine strains such as F9 and 255 [11–14]. Antigenic diversity plays a crucial role in vaccine ineffectiveness in clinical practice, underscoring the urgent need for updated vaccines that reflect the contemporary evolutionary profile of FCV [15, 16].

FCV is a positive‐strand, nonenveloped RNA virus [1]. Its genome, approximately 7.5 kb in size, is composed of three open reading frames (ORFs). ORF1 encodes nonstructural proteins, whereas ORF2 and ORF3 encode the major capsid protein VP1 and the minor structural protein VP2, respectively [17, 18]. VP1 is divided into six regions(A–F) according to its conserved sequences [19, 20], Among these, regions A, B, D, and F are relatively conserved across different FCV strains, whereas regions C and E exhibit significant variability [21]. The E region plays a crucial role in antigenic variation and receptor binding. Specifically, within the E region, the 5^′^ hypervariable region (5^′^HVR) contains essential linear B‐cell epitopes targeted by neutralizing antibodies, such as the extensively studied epitope that covers specific amino acids [21, 22]. Additionally, this region contains the binding site for the cellular receptor feline junctional adhesion molecule A (fJAM‐A), which mediates viral attachment and entry [23, 24].

The host range of FCV is not limited to domestic cats. Sporadic cases of FCV infection have been documented in various wild felids, including cheetahs, lions, and tigers [25–29]. The identification of the highly virulent tiger‐derived FCV strain TIG‐1, which causes 100% morbidity and mortality in experimentally infected cats, highlights its potential for cross‐species transmission and emphasizes the threat it poses to endangered wildlife populations [30]. The discovery of FCV‐like viruses in dogs adds complexity to the epidemiological landscape, indicating a broader host range than previously recognized [31, 32].

Extensive research has been conducted on FCV in domestic cats; however, significant knowledge gaps remain regarding the genetic diversity, evolutionary dynamics, and biological characteristics of FCV strains in endangered wild felids, including leopards and South China tigers. In this study, we isolated and characterized an FCV strain from captive South China tigers in a zoo. These findings are expected to enhance current understanding of the host range and genetic diversity of FCV and to provide crucial insights into the potential for cross‐species viral transmission and its implications for the conservation of these iconic endangered species.

## 2. Materials and Methods

### 2.1. Sample Collection

Tissue samples from the South China tiger were obtained at a wildlife conservation agency in Henan Province, China. All samples were collected by veterinarians following standard veterinary procedures and stored at −80°C for subsequent analysis.

### 2.2. Virus Isolation and Identification

Tissue samples (heart, liver, spleen, lung, kidney, stomach, and pancreas) were homogenized in Dulbecco’s modified Eagle’s medium (DMEM) supplemented with 2× penicillin‐streptomycin and centrifuged at 12,000 × *g* for 10 min at 4°C. The supernatant was filtered through a 0.22 μm membrane and inoculated onto monolayers of Crandell‐Rees feline kidney (CRFK) cells cultured in DMEM. Following 1 h of adsorption at 37°C in a 5% CO_2_ incubator, the inoculum was removed, and cells were washed twice with phosphate‐buffered saline (PBS). Fresh DMEM containing 10% fetal bovine serum (FBS) (Gibco, USA) was added, and the cells were incubated for 24 h to observe cytopathic effects (CPEs). Mock‐infected CRFK cells served as controls. The cultures were subjected to three freeze–thaw cycles, followed by centrifugation at 4000 × *g* for 10 min, and the supernatants were collected and stored at −80°C. The harvested cell cultures were blindly passaged five consecutive times, and the isolated virus was named FCV LYH1224.

### 2.3. Metatranscriptomic Screening for Coinfecting Pathogens

To evaluate the potential involvement of other pathogens in the disease process, we conducted a retrospective metatranscriptomic screening of the sequencing dataset (BioProject accession number PRJNA1479210). Raw reads were first quality‐filtered using fastp v0.23.2 to obtain clean reads. For bacterial detection, clean reads were profiled against a panel of bacterial marker genes using MetaPhlAn v4.0.6. For comprehensive pathogen discovery, clean reads were assembled de novo using MEGAHIT v1.2.9 into contiguous sequences (contigs), which were then aligned against the NCBI nonredundant (nr) protein database, the Fungi ITS RefSeq database, and the Midori2 database (containing curated sequences from diverse eukaryotic microorganisms) to identify viral, fungal, and parasitic sequences.

### 2.4. Immunofluorescence Assay (IFA)

CRFK cells infected with the FCV strain were incubated for 12 h, washed once with PBS, and fixed with 4% paraformaldehyde for 30 min at room temperature. After three washes with PBST, the cells were incubated with a mouse monoclonal antibody against the FCV VP1 protein for 1 h at 37°C. Following three additional washes with PBST, the cells were incubated with FITC‐conjugated goat anti‐mouse IgG (Beyotime, Wuhan, China) for 1 h at 37°C. Nuclei were counterstained with DAPI. Fluorescence signals were visualized using a fluorescence microscope (Nikon, Japan).

Formalin‐fixed, paraffin‐embedded (FFPE) kidney tissue samples were sectioned into 5 μm‐thick slices. Immunohistochemical (IHC) staining was performed using a mouse monoclonal antibody against the FCV VP1 protein. Briefly, tissue sections were deparaffinized, rehydrated, and rinsed with distilled water. After cooling, the sections were washed in Tris‐buffered saline (TBS) for 3 min, followed by immersion in 3% H_2_O_2_ for 30 min to block endogenous peroxidase activity. Nonspecific binding was blocked by incubation with 10% normal goat serum for 30 min at room temperature. The sections were then washed with TBS and incubated with the primary antibody for 1 h at room temperature. Subsequently, the sections were incubated with a goat anti‐mouse secondary antibody for 45 min at 37°C, washed three times with TBST, counterstained with hematoxylin for 1 min, rinsed, and differentiated in acid alcohol for 1–2 s. Images were captured using a white‐light microscope.

### 2.5. Transmission Electron Microscopy (TEM)

CRFK cells infected with the FCV LYH1224 strain were gently scraped and fixed in 2.5% glutaraldehyde in 0.1 M phosphate buffer (PBS, pH 7.4) at 4°C. After rinsing with PBS, the specimens were post‐fixed with 1% osmium tetroxide for 2 h at room temperature, dehydrated through a graded ethanol series (30%–100%) and acetone, and infiltrated with epoxy resin 812. The samples were embedded in pure resin and polymerized at 60°C for 48 h. Semi‐thin sections (1.5 μm) were stained with toluidine blue for light microscopic targeting. Ultrathin sections (60–80 nm) were mounted on copper grids, stained with 2% uranyl acetate and 2.6% lead citrate, and examined under a transmission electron microscope.

### 2.6. Real Time RT‐PCR (RT‐Quantitative PCR [qPCR]) for FCV Analysis

Total RNA was extracted from tissue homogenate supernatants using the AxyPrep Body Fluid Viral DNA/RNA Miniprep Kit (AXYGEN, USA) according to the manufacturer’s instructions. For cDNA synthesis, 5 μg of total RNA was reverse transcribed in a 20 μL reaction volume using the PrimeScript IV 1st Strand cDNA Synthesis Mix (Takara). Specific primers targeting the conserved region of the FCV genome (Table S1) were used for quantification.

To determine absolute viral copy numbers, a standard curve was generated using a DNA fragment containing the known concentrations of the FCV VP1 gene target amplicon. The fragment was serially diluted 10‐fold from 10^8^ to 10^1^ copies/μL. qPCR was performed using the ChamQ Universal SYBR qPCR Master Mix (Vazyme, Nanjing, China) on a CFX Connect Real‐Time System (Bio‐Rad, Hercules, CA, USA). The thermal cycling protocol consisted of an initial pre‐denaturation at 98°C for 1 min, followed by 40 cycles of 95°C for 10 s, 60°C for 30 s, and 72°C for 20 s. Each dilution was tested in triplicate, and a standard curve was constructed by plotting the threshold cycle (Ct) values against the log_10_ of the copy numbers. The amplification efficiency and correlation coefficient (*R* ^2^) were calculated from the standard curve. Viral copy numbers in tissue samples were interpolated from the standard curve based on their Ct values and normalized to 100 ng of total RNA per reaction. Samples with Ct values beyond the linear range of the standard curve were reported to be below the detection limit. All reactions were performed in three independent biological replicates, and mean copy numbers were used for subsequent analyses.

### 2.7. Virus Titer Assay

The FCV LYH1224 strains were titrated using the 50% tissue culture infective dose (TCID_50_) assay in CRFK cells. Briefly, CRFK cells were seeded into 96‐well plates and cultured in DMEM supplemented with 5% FBS. After 24 h of incubation, the cells were inoculated with 10‐fold serial dilutions (10^−1^–10^−10^) of the virus strain and cultured at 37°C for 48–72 h, and TCID50 was calculated using the Reed–Muench method. All assays were performed in triplicate.

### 2.8. Replication Properties in CRFK Cells

For comparison, a reference strain, designated FCVGD0217, was included in the growth kinetics analysis. This strain was isolated from a domestic cat presenting with upper respiratory signs in Guangzhou, China, in 2024. It belongs to GI and has been passaged five times in CRFK cells. Its complete genome sequence has been deposited in GenBank under accession number PZ542403. Detailed information on all strains used in this study is provided in Table S2.

CRFK cells cultured in 6‐well plates were infected with FCV LYH1224 and FCV GD0217 at a multiplicity of infection (MOI) of 0.001. Infected cells and culture supernatants were harvested at 6, 12, 24, 36, 48, 60, and 72 h post‐infection (hpi) and subjected to three freeze–thaw cycles to release viral particles. The supernatants were collected and titrated by inoculating CRFK cells in 96‐well plates with 10‐fold serial dilutions (10^−1^–10^−10^); the plates were then incubated for an additional 24–48 h. CPEs were monitored, and the 50% tissue culture infective dose (TCID_50_) was calculated using the Reed–Muench method at each time point to generate growth curves. All assays were performed with three independent biological replicates. For statistical analysis, growth curve data were evaluated using a two‐way ANOVA with a mixed‐effects model, followed by Šidák’s multiple comparisons test at each time point. All statistical analyses were performed using GraphPad Prism 9.5 (GraphPad Software, San Diego, CA, USA).

### 2.9. Genome Sequencing and Phylogenetic Analysis

Viral RNA was extracted from cell culture supernatants of the FCV LYH1224 isolate using the AxyPrep Body Fluid Viral DNA/RNA Miniprep Kit (AXYGEN, USA). Libraries were sequenced on the Illumina NovaSeq 6000 platform (PE150, Illumina), and the resulting raw reads were assembled using MEGAHIT v1.2.9. The complete genome sequences were annotated using the NCBI ORFfinder tool. To obtain the complete gene, a total of three pairs of primers were designed for amplification (Table S1). Reference sequences were selected according to the following criteria: initially, known FCV genomes representing both GI and GII genotypes were included to ensure alignment with established lineage definitions [9, 15]; on this basis, we further incorporated all previously published wild felid‐derived strains [16, 30], as well as vaccine and reference strains commonly used in the literature. These criteria ensured a comprehensive representation of global FCV diversity. Multiple sequence alignments were conducted using MAFFT v7.313, and pairwise nucleotide and amino acid sequence identities were calculated using Geneious. For phylogenetic analysis, phylogenetic trees were constructed based on the full‐length genome and VP1 gene sequences using the maximum likelihood method with 1000 bootstrap replicates in IQ‐TREE software, and the best‐fit substitution models were determined by IQ‐TREE with ModelFinder and are specified as GTR + F+R5 for the genome tree and LG + F+R5 for the VP1 tree.

To investigate potential recombination events, recombination analysis was performed on the complete genome of FCV LYH1224 using RDP5 v5.5 software with the following detection methods: RDP, GENECONV, BootScan, MaxChi, Chimaera, SiScan, and 3Seq. A panel of nine reference strains was included in the analysis. Recombination events were considered significant only if they were supported by at least four of the seven methods and yielded a Bonferroni‐corrected *p*‐value of < 0.05.

### 2.10. Comparative Analysis of Key Amino Acid Sites in the VP1 Protein

To characterize the molecular features of the VP1 protein in the wildlife‐derived FCV LYH1224, the amino acid sequences were aligned with nine reference strains, including vaccine strain F9, virulent strain 2280, cheetah‐FCV strain, TIG‐1, DL38, FCV‐YH/16, FCV‐FB‐NJ13, and representative respiratory and enteric strains, using MegAlign (DNASTAR). Detailed information on all strains used in this study is provided in Table S2. The analysis focused on defined linear epitopes and nine significant virulence‐related sites.

## 3. Result

### 3.1. Clinical and Anatomical Characteristics of the Infected Tiger

A South China tiger died at an animal rescue center in Henan Province. Externally, the tiger had disheveled fur and was emaciated, with no external injuries observed on the limbs or trunk. Gross necropsy revealed extensive grayish‐white lesions and dark red hemorrhagic foci in the lungs, which had lost their normal spongy texture (Figure 1A). Pericardial effusion was observed within the pericardial cavity, and numerous blood clots were found in the ventricular lumen (Figure 1B). The liver was enlarged with scattered petechial hemorrhages (Figure 1C). The spleen showed mild petechial hemorrhages (Figure 1D). The gastrointestinal tract was devoid of ingesta, and scattered punctate hemorrhages were observed on the gastric mucosa (Figure 1E,F). Based on these findings, the tiger was diagnosed with respiratory failure secondary to severe pneumonia.

**Figure 1:**
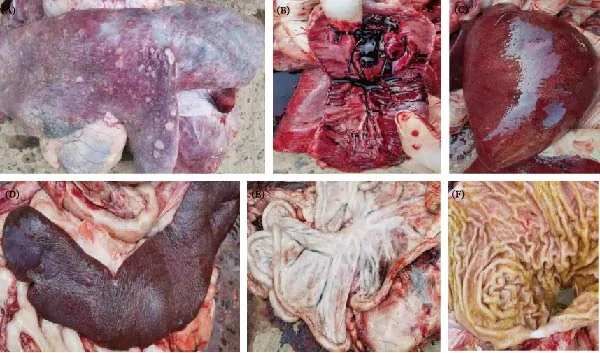
Pathological symptoms in the tiger infected with calicivirus. (A) Gross examination revealed extensive gray‐white foci and dark red hemorrhages in the lungs. (B) Pericardial effusion. (C) Hepatomegaly. (D) Mild petechial hemorrhage on the spleen. (E, F) Absence of ingesta in the gastrointestinal tract with scattered punctate hemorrhage on the gastric mucosa.

### 3.2. Kidney Damage and Viral Load in Tiger‐Derived LYH1224 Strain

Histopathological examination of the kidney from the deceased tiger revealed widespread degenerative changes in both the glomeruli and renal tubules. Renal tubular epithelial cells exhibited pyknosis, necrosis, and sloughing (black arrows), along with prominent proliferation of the interstitial connective tissue (green arrows) (Figure 2A). IHC analysis was performed to assess FCV VP1 antigen distribution in the kidney. Positive immunolabeling was predominantly localized within the cytoplasm of renal tubular epithelial cells. The positive signals were focally distributed, often clustering in the corticomedullary junction (Figure 2C). No specific labeling was observed in uninfected tiger kidney tissue sections (negative control) (Figure 2B), confirming the specificity of the IHC staining.

**Figure 2:**
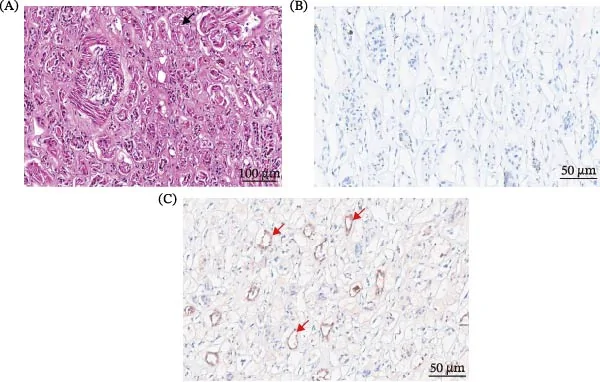
Histopathological sections and IHC from the kidney of the virus‐infected tiger. (A) Histological examination of the kidney revealed nuclear pyknosis, necrosis, and detachment of tubular epithelial cells (black arrows), and connective tissue hyperplasia (green arrows). (B, C) Immunohistochemical analysis using anti‐FCV VP1 protein antibody (brown) detected viral antigens in tiger kidney tissue. (B) Kidney tissue from an uninfected tiger (negative control). (C) Positive sample; red arrows indicate viral colonization.

### 3.3. Virus Isolation and Molecular Identification

Through metatranscriptomic sequencing and RT‐PCR validation, FCV nucleic acids were successfully detected in tissue samples collected from the South China tiger.

To assess the potential involvement of other pathogens in the disease process, we have performed a retrospective screening of the metatranscriptomic dataset for other common feline pathogens, including feline herpesvirus‐1 (FHV‐1), feline parvovirus (FPV), feline coronavirus (FCoV), feline immunodeficiency virus (FIV), feline bocavirus (FBoV), feline leukemia virus (FeLV), feline rotavirus (FRV), *Mycoplasma felis*, *Bordetella bronchiseptica* and *Chlamydia felis*, and *Salmonella*. No reads mapping to these pathogens were detected above background thresholds, suggesting that coinfection with these agents was unlikely in this case. Subsequently, the supernatant of positive tissue homogenates was inoculated into CRFK cells and blindly passaged to the fifth generation. The isolated FCV LYH1224 consistently produced typical CPEs, characterized by cell rounding, shrinkage, detachment, and the formation of grape‐like clusters, whereas mock‐infected cells did not show any cytopathy (Figure 3A). The isolate was identified by RT‐PCR using specific primers, and a 397 bp fragment was amplified from FCV LYH1224 (Figure 3B). An indirect IFA revealed specific green fluorescence in CRFK cells infected with the FCV LYH1224 strain, indicating successful expression of the viral VP1 protein (Figure 3C). The TCID_50_ of the FCV LYH1224 strain was determined to be 10^8.63^. TEM confirmed the presence of viral particles, which appeared as uniform 30–40 nm particles with dense cores (Figure 3D), confirming that the isolate was FCV.

**Figure 3:**
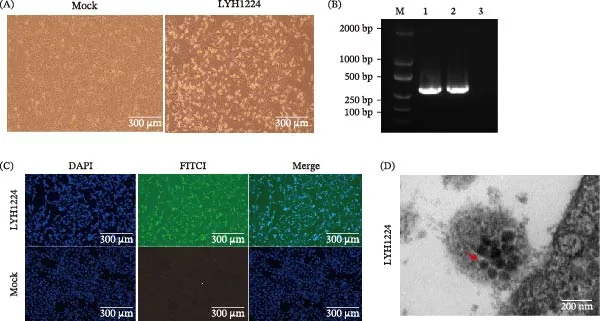
Isolation, identification, and analysis of the biological characteristics of the FCV LYH1224 strain. (A) CRFK cells were infected with FCV LYH1224 or control medium for 24 h. Mock‐infected cells served as a negative control. Scale bar = 300 μm. (B) RT‐PCR products were analyzed by 1% agarose gel. M: marker; Lanes 1–2: passages 1 and 5 of FCV LYH1224; Lane 3: negative sample. (C) IFA of CRFK cells infected with FCV LYH1224 for 24 h using anti‐FCV‐VP1 antibody. Uninfected cells served as a negative control. (D) Transmission electron microscopy of FCV LYH1224 particles (red arrows), approximately 30–40 nm in diameter.

### 3.4. Viral Growth Kinetics and Tissue Distribution

To evaluate the in vitro growth kinetics of the FCV LYH1224 isolate, CRFK cells were infected with FCV LYH1224 and FCV GD0217 (GenBank Number: PZ542403) at an MOI of 0.001, and viral titers were measured at 6, 12, 24, 36, 48, 60, and 72 hpi. As shown in Figure 4A, the peak yield of FCV LYH1224 was obtained at 36 hpi. During 6–36 hpi, FCV LYH1224 and FCV GD0217 exhibited similar replication kinetics. After 36 hpi, the titer of FCV LYH1224 gradually declined. In contrast, following a peak at 48 hpi, the titer of FCV GD0217 remained consistently higher than that of FCV LYH1224. These results indicate that FCV GD0217 has a stronger in vitro proliferative capacity and achieved a higher peak yield in CRFK cells, whereas the replication efficiency of FCV LYH1224 is relatively weaker, suggesting differences in replicative adaptability in a heterologous cell line among caliciviruses derived from different felid species.

**Figure 4:**
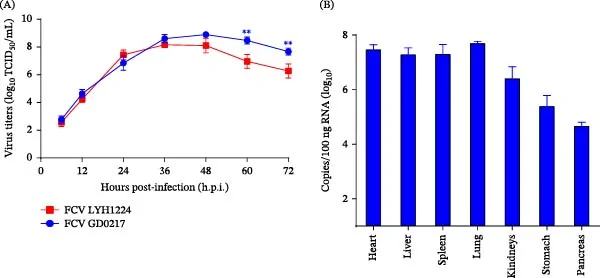
Growth kinetics and tissue tropism of FCV LYH1224. (A) Growth kinetics curve for FCV LYH1224 and FCV GD0217 (MOI = 0.001) in CRFK cells, constructed by measuring viral titers (TCID_50_) at 6, 12, 24, 36, 48, 60, and 72 hpi ( ^∗^ *p*  < 0.05;  ^∗∗^ *p*  < 0.01). (B) Tissue tropism of FCV LYH1224 evaluated by detecting VP1 gene expression across various tissues using qRT‐PCR.

To assess viral tissue tropism, viral loads were measured in clinically collected tissue samples from the South China tiger. High viral loads were detected in multiple tissues, including the heart, liver, spleen, lung, and kidney, whereas lower viral loads were detected in the stomach and pancreas (Figure 4B). These findings indicate that the FCV LYH1224 isolate exhibits broad tissue tropism.

### 3.5. Sequence Analysis

Through sequencing and assembly, the complete genome sequence of FCV LYH1224 (GenBank Number: PX960504) was determined to be 7764 bp. Sequence alignment confirmed that the transcriptome assembly sequence was consistent with the Sanger sequencing sequence. Comparative analysis was performed using the complete genome sequences of nine reference strains: F9 (attenuated vaccine strain), FCV‐2280 (highly virulent strain for inactivated vaccine), a previously reported cheetah strain, TIG‐1 (Tiger‐VSD‐FCV strain), DL38 (domestic cat nonvirulent strain), R‐GXLZ01‐19 (domestic cat respiratory symptoms strain), E‐GXNN04‐20 (domestic cat enteric symptoms strain), FCV‐FB‐NJ13 and FCV‐YH/16 (Tiger‐FCV strain), we performed a recombination analysis using RDP5 software (GENECONV, BootScan, MaxChi, Chimaera, SiScan, and 3Seq methods) on the complete genome of FCV LYH1224 against a panel of nine reference strains. No significant recombination events were detected. Whole‐genome sequence identity analysis revealed that FCV LYH1224 shared 76%–79.9% nucleotide identity with the nine reference strains; the identities with vaccine strains F9 and FCV‐2280 were 76.6% and 77.0%, respectively. Marked differences were also observed compared with previously reported tiger strains, showing 76.1% nucleotide identity with the respiratory strain R‐GXLZ01‐19 and the enteric strain E‐GXNN04‐20 (76%–76.5%) (Table 1).

**Table 1:** Comparative analysis of genomic sequences of the FCV LYH1224 isolate.

Strain	Tiger‐derived LYH1224				
	Genome	ORF1	ORF2	ORF3	VP1‐E
	nt	nt/aa	nt/aa	nt/aa	nt/aa
F9 strains	76.6	76.8/88.2	75/85.2	81.9/86.7	68.1/75.0
2280 strains	77.0	77.4/88.2	75.4/84.1	80.6/86.7	66.7/71.0
Cheetah strains	79.2	79.5/90.0	77.9/85.9	79.7/85.7	64.2/61.3
TIG‐1 strains	76.2	77.2/87.5	75.2/84.9	78.4/86.7	67.7/69.1
DL38 strains	79.9	80.4/91.7	78.3/87.9	82.5/91.4	69.5/68.8
R‐GXLZ01‐19 strains	76.0	77.2/88.4	75.4/85.3	77.5/86.7	68.8/76.3
E‐GXNN04‐20 strains	76.5	77.3/88.3	75.1/84.2	77.1/87.6	69.2/72.0
FCV‐YH/16	79.1	79.3/90.7	77.4/86.7	84.7/92.5	82.3/96.8
FCV‐FB‐NJ13	84.9	86.8/96.4	79.6/87.4	88.5/96.2	87.6/98.9

The ORF1 gene was 5295 bp in length, encoding a polyprotein of 1765 amino acids. Nucleotide and amino acid identities between FCV LYH1224 and the nine reference strains ranged from 76.8%–86.8% and 87.5%–96.4%, respectively. Notably, the highest amino acid identity was observed with FCV‐FB‐NJ13 (96.4%). The ORF2 gene was 2007 bp in length, encoding the VP1 capsid protein of 669 amino acids. Nucleotide and amino acid identities with the reference strains ranged from 75.1%–79.6% and 84.1%–87.9%, respectively. The ORF3 gene was 321 bp in length, encoding the VP2 protein of 107 amino acids. Nucleotide and amino acid identities between FCV LYH1224 and the reference strains ranged from 77.1%–88.5% and 85.7%–96.2%, respectively. The VP1‐E region, which spans 285 bp encoding 95 amino acids and represents the critical domain for interaction with the fJAM‐A receptor, exhibited the lowest identity among the ORFs analyzed, with nucleotide and amino acid identities ranging from 64.2% to 87.6% and 61.3% to 98.9%, respectively. The VP1‐E region is the most diverse region in the entire genome (Table 1).

### 3.6. Phylogenetic Analysis

Phylogenetic trees were constructed based on the complete genome and VP1 gene sequences (Figure 5). All FCV strains were classified into two genotypes: GI and GII. GI strains are globally distributed and remain predominant, whereas GII strains are predominantly identified in Asia.

**Figure 5:**
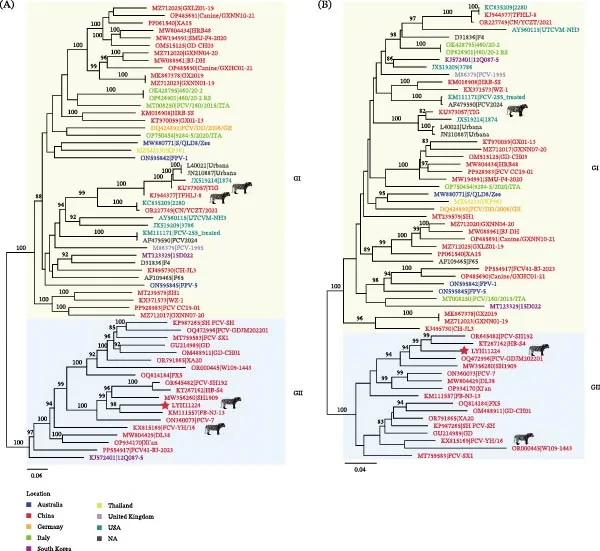
Maximum likelihood phylogenetic trees of FCV based on the nucleotide sequences of the complete genome and the VP1 genes. (A) complete genome. (B) VP1 genes. The statistical support of tree nodes was evaluated by bootstrapping method with 1000 replicates. The genotype GI is circled in yellow, and the genotype GII in blue. Different colors are used to label strains from different regions, and published tiger isolates are labeled with cartoon icons. Strain used in this study are denoted by 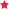.

Based on the complete genome phylogenetic tree, FCV LYH1224 clustered within the GII clade and was closely related to the FCV‐FB‐NJ13 strain isolated from a domestic cat in eastern China in 2013 (Figure 5A). In contrast, FCV LYH1224 was genetically distinct from previously reported tiger‐derived isolates, including TFHLJ‐8 and TIG‐1 (both GI genotype), but was more closely related to the tiger‐derived isolate FCV‐YH/16. Phylogenetic analysis based on the VP1 gene sequences also divided all FCV strains into GI and GII. GI strains were isolated from multiple countries across the Americas, Europe, and Asia, including Australia, China, Germany, Italy, South Korea, the United Kingdom, and the United States (Figure 5B). In contrast, GII strains have been exclusively isolated from Asia. FCV LYH1224 and the other three tiger‐derived FCV isolates are distributed across different branches of the phylogenetic tree and are all genetically distant from one another, suggesting considerable genetic diversity among FCV strains originating from wild animal hosts in different geographic regions. Importantly, FCV LYH1224 shows low genetic similarity to the vaccine strains F9 and 2280, with amino acid identities of 75% and 71% in the VP1‐E hypervariable region, respectively (Table 1).

### 3.7. Comparative Analysis of Key Amino Acid Sites in VP1 Protein

To elucidate the molecular characteristics of the VP1 protein in the FCV LYH1224 isolate, a comparative analysis of critical amino acid residues was conducted. Investigation of two well‐defined linear epitopes revealed distinct patterns of conservation. The non‐neutralizing epitope PAGDY (amino acids 431–435) demonstrated a high degree of conservation, whereas the neutralizing epitope spanning amino acids 445–451 exhibited considerable variability. Specifically, residues I445, T447, and Y451 were largely conserved, while positions 446, 448, 449, and 450 displayed notable heterogeneity. Of particular interest, unique amino acid substitutions, such as S448 and A449, were identified in isolates derived from wildlife, including FCV LYH1224 (Table 2), indicating potential antigenic divergence within these strains.

**Table 2:** Comparison of key amino acid differences in the E domain of the FCV VP1 protein.

Strain	Non‐neutralizing epitope	Neutralizing epitope	Virulence‐associated sites							
	431–435	445–451	438	440	448	452	455	465	492	
F9 strains	PAGDY	ITTATGY	T	G	A	T	D	G	L	
2280 strains	PAGNY	IATATEY	T	S	A	H	D	S	K	
Cheetah strains	PAGEY	IATAVDY	V	Q	A	T	T	S	L	
TIG‐1 strains	PAGDY	ITTASQY	T	S	A	T	D	G	V	
DL38 strains	PAGDY	IETREDY	T	P	R	T	T	G	L	
R‐GXLZ01−19 strains	PAGDY	IVTAREY	T	G	A	S	D	S	V	
E‐GXNN04−20 strains	PAGDY	ITTAKEY	T	G	A	S	G	S	V	
FCV‐YH/16	PSGDY	ITTSSEY	T	Q	S	D	T	S	L	
FCV‐FB‐NJ13	PAGDY	ITTAMEY	T	Q	A	D	T	S	L	
FCV LYH1224	PAGDY	ISTAKEY	T	G	A	A	D	G	I	

Further comparison of nine residues previously implicated in the differentiation of the virulent systemic disease (VSD) pathotype revealed significant variation. Relative to the vaccine strains F9 and 2280, the FCV LYH1224 isolate exhibited unique substitutions at positions 452A and 492I (Table 2). When contrasted with previously characterized Tig‐1 strains, FCV LYH1224 showed divergence at three of the nine residues: S440G, T452A, and V492I. At the other four positions (T438, A448, D455, and G465), FCV LYH1224 shared residues with TIG‐1. Notably, the concurrent presence of mutations at positions 452A and 492I in FCV LYH1224 was not observed in any reference strains, including known VSD isolates. These findings suggest that FCV strains circulating in wild felids may have undergone host‐specific adaptation, resulting in unique genetic signatures.

## 4. Discussion

FCV is a well‐known virus that is widespread among domestic cats, causing illnesses that range from mild upper respiratory infections to deadly VSD [3, 33]. While occasional cases have been reported in wild cats, little is known about the genetic variation, evolutionary patterns, and biological traits of FCV strains in endangered species like the South China tiger [34–36]. In this research, we successfully isolated a new FCV strain, named FCV LYH1224, from a captive South China tiger that died from severe pneumonia and systemic infection. Detailed genomic and biological analysis of this strain offers valuable understanding of FCV’s host range, genetic diversity, and adaptive evolution in endangered wild felines, which is crucial for conservation efforts and vaccine development.

The successful isolation of FCV from a South China tiger confirms that these endangered species are susceptible to FCV infection. Previous studies have documented FCV infection in other wild felids, including lions, cheetahs, and Siberian tigers [25–27, 30]. However, this is the first report of FCV isolation and complete genomic characterization of the South China tiger. Viral RNA was detected in multiple tissues, including the heart, liver, spleen, lung, and kidney, indicating systemic dissemination, consistent with the pathogenic potential of virulent FCV strains [5]. Gross necropsy findings revealed extensive pulmonary lesions, hepatic abnormalities, and gastrointestinal hemorrhage, which were consistent with characteristics of VSD FCV infection [6, 30]. While coinfection was not detected, the possibility of unidentified pathogens or noninfectious causes cannot be excluded. The widespread viral distribution and severe organ pathology suggest that FCV may have contributed substantially to the fatal outcome, although a definitive causal relationship cannot be established from a single case. These findings underscore the potential threat of FCV to captive endangered felid populations and highlight the need for active surveillance in wildlife rescue and zoo facilities.

Comparative in vitro growth kinetics showed that the tiger‐derived FCV LYH1224 and a domestic cat‐derived FCV‐GD0217 strain exhibited similar replication patterns in CRFK cells, but with notable differences. FCV LYH1224 reached its peak titer earlier (36 versus 48 hpi) and then declined more rapidly, whereas FCV‐GD0217 maintained a higher sustained titer. The ability of this wildlife‐derived strain to readily infect CRFK cells implies that it retains the capacity to infect domestic cats, raising concerns about bidirectional cross‐species transmission at the domestic–wildlife interface [26]. Such transmission events could pose serious risks to both vulnerable wild populations and domestic animal health.

Phylogenetic analysis based on the complete genome and VP1 gene unambiguously classified FCV LYH1224 as GII, a clade predominantly circulating in Asia [9, 37]. However, it formed a distinct branch separate from previously reported Siberian tiger isolates, suggesting species‐specific and complex genetic backgrounds among FCV strains from different wildlife sources. Notably, FCV LYH1224 was most closely related to the domestic cat strains FCV‐FB‐NJ13 and FCV‐SH1909, both isolated in eastern China in 2013 and 2019, respectively. This phylogeographic clustering suggests the possibility of cross‐species transmission from domestic cats to tigers. Stray cats have been occasionally observed near the enclosures of infected South China tigers, offering a plausible route of viral introduction, although none were tested during this outbreak. This scenario is supported by a well‐documented outbreak of VS‐FCV in a zoological setting, where a stray cat live‐trapped outside the feline building was confirmed as the infection source, and sequencing revealed identical FCV isolates from the stray cat, affected tiger cubs, and a lion [25]. In that outbreak, a 15‐day‐old tiger cub died from extensive tongue necrosis and systemic dissemination, illustrating the severe pathogenic potential of FCV in neonatal tigers. Given that FCV can persist on dry surfaces at room temperature for up to 28 days, indirect transmission via fomites is also plausible. Collectively, these observations support the hypothesis that a domestic cat‐derived FCV lineage may have been introduced into the tiger population, consistent with the phylogenetic clustering pattern observed in our study [38] This temporal and geographic clustering suggests that certain FCV lineages may persist and evolve within specific regions over long periods [39, 40]. A significant genetic distance was observed between the FCV LYH1224 isolate and the vaccine strains F9 and 2280, with amino acid identities of only 75% and 71% in the VP1‐E hypervariable region, respectively (Table 1). This finding is consistent with previous reports indicating that the rapid evolution of FCV may compromise the protective efficacy of current commercial vaccines against emerging field strains [14–16]. Consequently, this poses a considerable challenge for the prevention and control of FCV in captive felids in China and underscores the urgent need for the development of novel, broad‐spectrum, and highly efficacious vaccines.

Comparative analysis of the VP1 protein revealed unique amino acid signatures in key antigenic regions of the FCV LYH1224 strain. The non‐neutralizing epitope PAGDY (aa 431–435) was highly conserved across all strains analyzed, consistent with previous findings by Cubillos‐Zapata et al. [22] and Lu et al. [23], which demonstrated that residues within this epitope are critical for capsid assembly and viral viability. The 100% conservation of P431, G433, and Y435 highlights their critical structural function. Therefore, monoclonal antibodies targeting this epitope may represent broadly cross‐reactive diagnostic tools for FCV detection across diverse hosts [22]. In contrast, the neutralizing epitope ITTANQY (aa 445–451) exhibited marked variability in the wildlife isolates. The unique amino acid substitutions identified in FCV LYH1224 (S446) suggest antigenic divergence from domestic cat strains. This finding indicates that neutralizing antibodies induced by conventional GI‐based vaccines may exhibit reduced binding affinity to this wildlife‐derived strain.

Several limitations of this study should be acknowledged. First, this study is based on a single individual, and it remains unknown whether the genetic features of FCV LYH1224 are representative of broader South China tiger populations. Second, although the clinical and pathological findings strongly suggest a pathogenic role for FCV, the exact contribution of the virus to the animal’s death cannot be definitively established due to the absence of controlled experimental data and the potential involvement of other concurrent pathogens. Third, the functional significance of the identified unique amino acid substitutions has not been experimentally validated. Future research should focus on large‐scale surveillance in free‐ranging and captive felids, functional characterization of key mutations using reverse genetics, and development of region‐specific or wildlife‐targeted vaccine candidates.

## 5. Conclusion

In summary, this study provides the first comprehensive characterization of an FCV strain from a South China tiger, demonstrating that it belongs to GII, possesses unique genetic signatures in critical antigenic and virulence‐associated regions, and exhibits broad tissue tropism. Given its considerable divergence from vaccine strains, current vaccination protocols for captive felids may require reassessment, though confirmation through larger‐scale studies is essential. As a preliminary single‐case report, these findings provide a valuable basis for future surveillance and functional investigations to evaluate whether the observed genetic traits are representative of broader populations and to guide evidence‐based vaccine strategies.

## Author Contributions

Wu Chen and YuShan Ding conceived and designed the experiments. YuShan Ding and Zhaoyang Liu did the data curation, investigation, methodology, and writing – original draft. Wu Chen, Niu Zhou, Chen Wang, Yajiang Wu, Heng Liu, Tianping Li, Xuanjiao Chen, Weijun Xie, Minting Liu, Shiming Peng, Fen Shan and Xinyi Cao collected the sample. Hai Wang did the evolutionary analysis and visualization. Wu Chen and Yongyi Shen contributed to funding acquisition. Wu Chen, Fen Shan, Minting Liu, Mengna Lv and Zujin Chen were involved in project administration and supervision. Wu Chen and Yongyi Shen did writing – review and editing.

## Funding

This study was supported by the Guangzhou National Research Laboratory (Grant GZNL2023A01001), the National Natural Science Foundation of China (Grant U24A20363 to Yongyi Shen), Guangdong Provincial Key R&D Program (Grant 2022B1111040001 to Yongyi Shen), and Guangdong Major Project of Basic and Applied Basic Research (Grant 2020B0301030007 to Yongyi Shen).

## Disclosure

All authors have read and agreed to the published version of the manuscript.

## Ethics Statement

No laboratory animals were involved in this study. The study protocol was reviewed and approved by the Animal Ethics and Management Committee of Guangzhou Zoo and Guangzhou Wildlife Research Center (Permit Number GZZOO202401).

## Conflicts of Interest

The authors declare no conflicts of interest.

## Supporting Information

Additional supporting information can be found online in the Supporting Information section.

## Supporting information


**Supporting Information** Table S1. Primers used for complete genome amplification and detection of feline calicivirus (FCV) in this study. The table lists primer names, sequences (5^′^→3^′^), genomic positions, and amplicon sizes. Table S2. Summary of reference strains used in this study. The table includes strain name, GenBank accession number, host species, geographic origin, year of isolation, genotype, and pathotype (where available) for all reference strains included in the phylogenetic and sequence identity analyses.

## Data Availability

The raw transcriptome sequencing data generated in this study have been deposited in the NCBI database under BioProject Accession Number PRJNA1479210 and are available at https://www.ncbi.nlm.nih.gov/bioproject/PRJNA1479210.
