# Supplementary material for: Genetic Diversity and Biological Characteristics of a Novel Feline Calicivirus Strain Isolated From Zoo‐Captive South China Tiger (Panthera tigris amoyensis)
Source: Transbound Emerg Dis. 2026 Oct 9;2026:4900253. doi: 10.1155/tbed/4900253 (PMC13650507; doi:10.1155/tbed/4900253)
Supplement: Supplementary file 1 [file TBED-2026-4900253-s001.docx]

| Supplementary Table 1. The primers used in this study. | | | |
| --- | --- | --- | --- |
| Primer S | Sequence (5′-3′) | Position | Amplicon Size |
| FCV-Tig-F1 | ATGCAGTTACTCTGCCATTGTC | 1-22 | 2493 bp |
| FCV-Tig-R1 | CAGGTGCCGGCGAAGAGCCCA | 2495-2515 |  |
| FCV-Tig-F2 | CCAATCAACATGTGGTAACTGT | 2451-2472 | 2736 bp |
| FCV-Tig-R2 | TGTTATAATTTGGAGGGTCAA | 5208-5228 |  |
| FCV-Tig-F3 | GGTTGAGTTCTACAACAAAGTTC | 5143-5166 | 2597 bp |
| FCV-Tig-R3 | ATCTTAGGATTGATAAACACTG | 7742-7763 |  |
| TY-Tig-FCV-F | GAATTGGCTAAGATCTTGCATGA | 1313-1335 | 397bp |
| TY-Tig-FCV-R | TATCACAATTTAAAACCATAGG | 1688-1709 |  |
| qPCR-FCV-F | GGRAARATTGTCAATGAHARBGT | 2338-2390 | 163bp |
| qPCR-FCV-R | GGCAGTAAGCACATCATATGC | 2509-2529 |  |
| qPCR-GAPDH-qF | AGAGCTGAATGGGAAACTCACTG |  | 133bp |
| qPCR-GAPDH-qR | TGATGCCTGTTTCACCACCTTC |  |  |

Supplementary Table 2. Information on reference strains used in the study.

| Viral strain | GenBank | Host | Country | Year | | Genotype | Pathotype |
| --- | --- | --- | --- | --- | --- | --- | --- |
| F9 | M86379 | Felis catus | UK | | 1992 | I | Vaccine strains |
| 2280 | KC835209 | Felis catus | USA | | 2013 | I | Vaccine strains |
| Cheetah strain | GU214989 | Cheetah | China | | 2004 | I | NA ^a^ |
| TIG-1 | KU373057 | Tiger | China | | 2015 | I | VSD strains ^b^ |
| DL38 | MW804429 | Felis catus | China | | 2021 | II | Nonvirulent strain. |
| R-GXLZ01-19 | MZ712025 | Felis catus | China | | 2019 | I | ORD strains ^c^ |
| E-GXNN04-20 | MZ712020 | Felis catus | China | | 2022 | I | Enteric symptoms strains |
| FCV-YH/16 | KX815169 | Tiger | China | | 2016 | II | NA |
| FCV-FB-NJ13 | KM111557 | Felis catus | China | | 2013 | II | ORD strains |
| FCV-GD0217 | PZ542403 | Felis catus | China | | 2024 | I | ORD strains |
| FCV‑SH1909 | MW356260 | Felis catus | China | | 2019 | II | NA |

a.NA:There are no studies on pathogenicity. b. VSD :Viral Systemic Disease c. ORD :Oral Respiratory Disease.
